# Daily Folic Acid and/or Vitamin B12 Supplementation Between 6 and 30 Months of Age and Cardiometabolic Risk Markers After 6–7 Years: A Follow-Up of a Randomized Controlled Trial

**DOI:** 10.1016/j.tjnut.2023.03.003

**Published:** 2023-03-06

**Authors:** Rukman Manapurath, Tor A. Strand, Ranadip Chowdhury, Ingrid Kvestad, Chittaranjan S. Yajnik, Nita Bhandari, Sunita Taneja

**Affiliations:** 1 Centre for Health Research and Development, Society for Applied Studies, Delhi, India; 2 Centre for International Health, University of Bergen, Norway; 3 Department of Research, Innlandet Hospital Trust, Lillehammer, Norway; 4 Regional Centre for Child and Youth Mental Health and Child Welfare, West, Norwegian Research Centre, Bergen, Norway; 5 Diabetes Unit, King Edward Memorial Hospital and Research Centre, Pune, India

**Keywords:** B vitamins, metabolic markers, homocysteine, cardiovascular diseases, young children

## Abstract

**Background:**

Deficiencies of vitamin B12 and folate are associated with elevated concentrations of metabolic markers related to CVDs.

**Objectives:**

We investigated the effect of supplementation of vitamin B12 with or without folic acid for 6 mo in early childhood on cardiometabolic risk markers after 6–7 y.

**Methods:**

This is a follow-up study of a 2 × 2 factorial, double-blind, randomized controlled trial of vitamin B12 and/or folic acid supplementation in 6–30-mo-old children. The supplement contained 1.8 μg of vitamin B12, 150 μg of folic acid, or both, constituting >1 AI or recommended daily allowances for a period of 6 mo. Enrolled children were contacted again after 6 y (September 2016–November 2017), and plasma concentrations of tHcy, leptin, high molecular weight adiponectin, and total adiponectin were measured (*N* = 791).

**Results:**

At baseline, 32% of children had a deficiency of either vitamin B12 (<200 pmol/L) or folate (<7.5 nmol/L). Combined supplementation of vitamin B12 and folic acid resulted in 1.19 μmol/L (95% CI: 0.09; 2.30 μmol/L) lower tHcy concentration 6 y later compared to placebo. We also found that vitamin B12 supplementation was associated with a lower leptin–adiponectin ratio in subgroups based on their nutritional status.

**Conclusions:**

Supplementation with vitamin B12 and folic acid in early childhood was associated with a decrease in plasma tHcy concentrations after 6 y. The results of our study provide some evidence of persistent beneficial metabolic effects of vitamin B12 and folic acid supplementation in impoverished populations.

The original trial was registered at www.clinicaltrials.gov as NCT00717730, and the follow-up study at www.ctri.nic.in as CTRI/2016/11/007494.

## Introduction

CVDs account for 32% of all deaths globally, and >3 quarters of these deaths occur in low- and middle-income countries (LMICs) [1].

Although clinical manifestations of CVD become evident in the adulthood [2], its pathogenesis begins in early life [[3], [4], [5], [6]]. Hence, it will be useful to start preventive measures during this critical window to reduce the burden of CVDs in LMICs.

Vitamin B12 and folate deficiencies are common in LMICs and are associated with elevated concentrations of cardiometabolic risk markers, predominantly homocysteine, which is related to CVDs among adults [[7], [8], [9]]. These vitamins are essential cofactors for converting homocysteine to methionine [[10], [11], [12]]. Inadequate nutritional intake and subsequent deficiencies result in elevated plasma tHcy concentrations [13]. It is well established that elevated homocysteine concentration due to inborn errors of metabolism leads to vascular dysfunction [14]. Several observational studies have found associations between high tHcy concentrations and premature cardiovascular disease risk in children and adolescents [15,16]. Randomized controlled trials (RCTs) among healthy adults, however, found no effects of interventions for lowering homocysteine on CVD outcomes [[17], [18], [19]].

In addition to tHcy, 2 important adipokines, that is, leptin and adiponectin, are associated with increased cardiometabolic risk at a young age [20,21]. Deficiencies of vitamin B12 or folate may disrupt the expression of adipokines, which could lead to an increased risk of CVDs [[20], [21], [22]]. Supplementation of vitamin B12 and folic acid decreased leptin and increased adiponectin concentrations in animals [23]. This effect has not been demonstrated in humans.

We conducted a double-blind, RCT of folic acid and vitamin B12 supplementation in urban neighborhoods of Tigri and Dakshinpuri in New Delhi, India, to assess whether daily administration of B vitamins reduced risk of common infections [24]. We followed-up these children to measure growth, neurodevelopment, and cognitive functioning after 6–7 y [25,26]. The effect on these outcomes has been reported previously. In this article, we present the long-term effect of B-vitamin supplementation in early childhood on cardiometabolic risk markers such as tHcy, leptin, and total and high molecular weight (HMW) adiponectin after 6–7 y.

## Methods

### Study setting, design, and participants

The children in the follow-up study had previously participated in a 2 × 2 factorial randomized, double-blind, placebo-controlled trial (CTRI/2016/11/007494) conducted in urban neighborhoods of Tigri and Dakshinpuri in New Delhi, India. One thousand children aged 6–30 mo were recruited in the main trial from January 2010 to September 2011. Detailed information on the design and sampling for the study has been published elsewhere [25,26]. Approximately 6 y later (September 2016 to November 2017), families were contacted and invited to participate in the follow-up study. Those who consented were enrolled. Weight (Digitron scales, to the nearest 50 g), length (locally manufactured infantometers, to the nearest 0.1 cm), and height (Seca 213, to the nearest 0.1 cm) were measured at the ages of 6–30 mo in the original study and at the ages of 6–9 y in the follow-up study. A 3-mL blood sample was taken for the measurements of tHcy, leptin, total and HMW adiponectin, the leptin/total adiponectin ratio (LAR), and leptin/HMW adiponectin ratio (LHMWR).

### Study interventions

In the original trial, children were randomly assigned in a 1:1:1:1 ratio in blocks of 16 to receive vitamin B12 and folic acid, vitamin B12, folic acid, or placebo supplements daily for 6 mo. The intervention was a lipid-based nutritional supplement prepared by Nutriset Ltd provided in jars prelabeled with the subject identification number. Children were supplemented with 1 spoon (5 g) if they were 6–11 mo, and 2 spoons (10 g each) if they were >11 mo of age.

The supplement contained 54.1 kcal of total energy, 0.7 g of protein, and 3.3 g of fat. For the groups that were assigned to receive B vitamins, the supplement also contained 1.8 μg of vitamin B12, 150 μg of folic acid, or both, constituting >1 AI (6–12 mo) or recommended daily allowances (1–3 y) [24,27]. Fieldworkers gave the supplements to the participants daily for 6 mo, except on Sundays and public holidays when the mothers did.

### Laboratory analysis

Three milliliters of blood was collected in EDTA-containing vacutainers (BD) for the assessment of biomarkers tHcy, leptin, total, and HMW adiponectin. The blood samples were collected at the field site and were kept on ice blocks after collection until they were transferred to the laboratory situated within the study area. The samples were centrifuged within 4 h of collection. The specimen was centrifuged (Remi Sales & Engineering Ltd) at ∼450× *g* at room temperature for 10 min in the field office. Plasma was separated and aliquoted into cryovials, and stored at −20 °C at the central laboratory until further analysis [25]. The samples were then shipped on dry ice to KEM Hospital Research Centre laboratory, Pune, India, for analysis. tHcy was analyzed by using commercial kits from Abbott Laboratories [27]. Leptin was measured by an ELISA (Alpco, Cat # 11-LEPHU-E01), and adiponectin was assayed by ELISA (both total and HMW; Alpco Diagnostics). tHcy was measured in all children enrolled in the follow-up study, whereas leptin, total, and HMW adiponectin were measured in a randomly selected subsample of 274 children due to limited funding.

## Ethics approval and consent to participate

The follow-up study was approved by the Ethics Committee of the Society for Applied Studies, Delhi, India, and the Norwegian Regional Committee for Medical and Health Research Ethics (REK 2014/1359). Written informed consent was obtained from the parents of all enrolled infants.

### Statistical analysis

The analysis was carried out using Stata version 16.1 [28]. Distribution normality was assessed via visual inspection of the histogram and with Shapiro–Wilk tests. We present the mean (SD) and median (IQR) values for the biomarkers of interest among those assigned to receive vitamin B12, folic acid, and vitamin B12 and/or folic acid against the placebo. The factorial design of the trial allowed us to evaluate both the individual and combined effects of the interventions on the outcomes. Given that our total sample size of children was 791, we had ≥80% power to detect ≥0.2 SD (1.1 μmol/L) difference in mean tHcy concentration between the 2 groups. Furthermore, for other outcomes such as leptin and adiponectin, we had 82% statistical power to detect a difference of 0.35 SD. These calculations assumed an alpha of 0.05.

We used generalized linear models of the Gaussian family with an identity link function to calculate crude and adjusted mean differences (95% CIs) between the intervention groups. The variables listed in Table 1 were initially assessed in the crude models. The variables that were significant at a 0.2 level were included in the adjusted models. Each of the variables that were not associated with the outcome in the crude models was then included one at a time and retained if significant at the 0.2 level. We included interaction terms to measure whether the 2 vitamins modified the effect of each other. We also explored the effects of folic acid supplementation (with or without vitamin B-12) or B12 supplementation (with or without folic acid) on all outcomes and on various prespecified subgroups such as stunting (<−2 z scores height/length-for-age), wasting (<−2 z scores weight-for-height/length), and underweight (<−2 z scores weight-for-age) at the baseline of the main trial. The placebo group was used as the reference category in all these comparisons.

**TABLE 1:** Baseline characteristics of the children enrolled in the main study and in the part of the follow-up study

Variables	All participants				Subsample			
	Follow-up study							
	Placebo (*n* = 200)	Vitamin B12 only (*n* = 193)	Folic acid only (*n* = 203)	Vitamin B12 and folic acid (*n* = 180)	Placebo (*n* = 67)	Vitamin B12 only (*n* = 67)	Folic acid only (*n* = 70)	Vitamin B12 + folic acid (*n* = 70)
Age, mo at baseline^1^, mean (SD)	16.28 (7.02)	15.94 (6.92)	16.37 (7.22)	16 (7.02)	16 (7.36)	15.61 (6.74)	16.87 (6.62)	15.23 (6.61)
Age, in y at follow-up, mean (SD)	7.38 (0.68)	7.38 (0.70)	7.39 (0.73)	7.41 (0.73)	7.45 (0.68)	7.45 (0.66)	7.57 (0.69)	7.39 (0.73)
Girls, *n* (%)	91 (45.05)	110 (54.73)	99 (48.53)	81 (44.02)	30 (44.78)	40 (59.70)	42 (60)	32 (45.71)
Wasted^1^, *n* (%)	23 (11.39)	27 (13.43)	21 (10.29)	19 (10.33)	8 (11.94)	9 (13.43)	6 (8.57)	8 (11.43)
Stunted^1^, *n* (%)	79 (39.11)	76 (37.81)	80 (39.22)	61 (33.15)	27 (40.30)	18 (26.87)	23 (32.86)	26 (37.14)
Underweight^1^, *n* (%)	65 (32.18)	65 (32.34)	65 (31.86)	52 (28.26)	22 (32.84)	12 (17.91)	23 (32.86)	21 (30)
BMI^1^, mean (SD)	14.33 (1.79)	14.26 (1.67)	14.31 (1.57)	14.22 (1.55)	14.52 (2.13)	14.46 (1.86)	14.41 (1.66)	14.07 (1.56)
Family size, *n* (%)								
Nuclear	113 (55.90)	123 (61.20)	118 (59.80)	110 (59.8)	39 (58.21)	42 (62.69)	41 (58.57)	40 (57.14)
Joint	89 (44.06)	78 (38.8)	86 (42.2)	74 (40.2)	28 (41.79)	25 (37.31)	29 (41.43)	30 (42.86)
Age of mother, mean (SD)	32.46 (9.59)	32.76 (9.64)	31.87 (6.63)	31.52 (4.61)	33.80 (12.69)	33.16 (12.33)	32.38 (9.20)	31.32 (5.34)
Socioeconomic status, *n* (%)								
Poorest	42 (20.79)	42 (20.90)	39 (19.12)	36 (19.57)	8 (11.94)	5 (7.46)	11 (15.71)	9 (12.86)
Very poor	43 (21.29)	51 (25.37)	34 (16.67)	30 (16.30)	10 (14.93)	15 (22.39)	8 (11.43)	12 (17.14)
Poor	37 (18.32)	42 (20.90)	35 (17.16)	44 (23.91)	12 (17.91)	12 (17.91)	10 (14.29)	12 (17.14)
Less poor	44 (21.78)	30 (14.93)	50 (24.51)	34 (18.48)	24 (35.82)	17 (25.37)	24 (34.29)	15 (21.43)
Least poor	36 (17.82)	36 (17.91)	46 (22.55)	40 (21.74)	13 (19.40)	18 (26.87)	17 (24.29)	22 (31.43)
Mother employed, *n* (%)	31 (15.35)	45 (22.39)	25 (12.25)	39 (21.20)	6 (8.96)	15 (22.39)	8 (11.43)	16 (22.86)
Ever breastfed (yes), *n* (%)	201 (99.5)	199 (99)	201 (98.53)	182 (98.9)	67 (100)	67 (100)	69 (98.57)	70 (100)
Child school, *n* (%)	107 (54.04)	116 (58.80)	137 (63.89)	115 (63.89)	46 (68.66)	45 (67.16)	52 (74.29)	49 (70)
Government								
Private	91 (45.96)	81 (41.12)	65 (36.11)	65 (36.11)	20 (29.85)	22 (32.84)	16 (22.86)	19 (27.14)
Plasma vitamin B12 (pmol/L), mean (SD)	296.5 (166.5)	317 (202.1)	309.6 (199.4)	330.2 (195.1)	284.9 (152.8)	339.4 (215.8)	329.6 (221.6)	328.2 (190.7)
Plasma folate (nmol/L), mean (SD)	15.2 (12.9)	15.2 (13.8)	16.1 (14.6)	15.3 (14.2)	15.9 (12.7)	14.2 (12.7)	18.7 (17.4)	17.5 (16.1)

1 Values are baseline values at the time of original trial.

## Results

**FIGURE 1:**
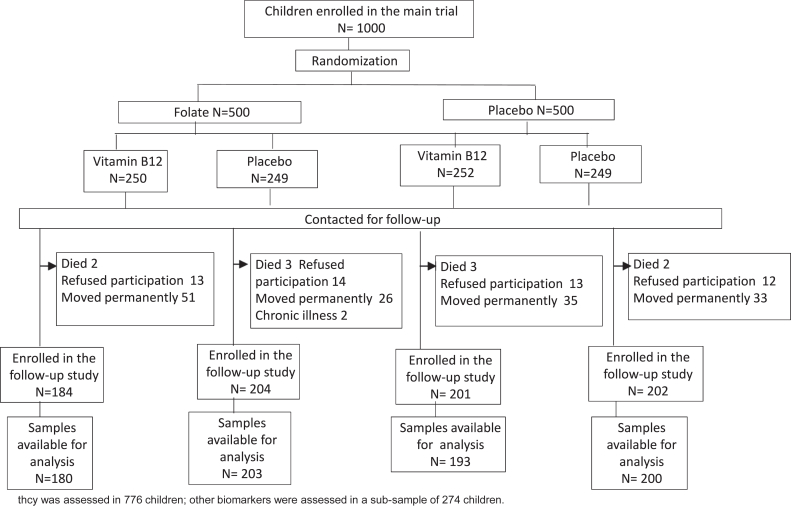
Flow of participants who were analyzed.

The participants enrolled in the study are shown in Figure 1. The enrollment in the follow-up study was from September 2016 to November 2017. Of the 1000 enrolled in the main study, 10 died before the start of the follow-up. Fifty-two parents did not consent for participation, and families of 145 children had moved permanently out of the study area. A total of 791 infants were re-enrolled in the follow-up study. We were able to collect and analyze blood samples for tHcy from 776 children. Leptin, total, and HMW adiponectin were assessed in a subsample of 274 children.

Table 1 shows the baseline characteristics of the enrolled children in the follow-up study along with the characteristics of those children selected for subsample analysis in the follow-up study. Demographic characteristics of the children in the follow-up sample and the subsample between the 3 intervention groups and the control group were similar. The overall mean (SD) age of children was 7.4 (0.7) ranging from 6 to 9 y. Fifty-six percent of the participants lived in nuclear families and the remaining in joint families. The proportion of infants stunted at enrollment was 39.1% in the placebo, 37.8% in B12, 39.2% in folic acid, and 33.2 % in the vitamin B12 and folic acid group. The proportion of wasting was 11.4%, 13.4%, 10.3%, and 10.3% among the placebo, vitamin B12, folic acid, and B12 and/or folic acid groups, respectively. Approximately one-third of the children had a vitamin B12 concentration of below 200 pmol/L, and close to one-third had a baseline folate concentration lower than 7.5 nmol/L [25].

The cardiometabolic risk markers by the 3 intervention groups and the placebo group are given in Supplemental Table 1.

Table 2 shows the effects of daily vitamin B12 and/or folic acid supplementation on cardiometabolic risk markers after 6–7 y in crude models and in models adjusted for baseline BMI of the child. Compared to placebo, the concentration of tHcy was lower among children that received vitamin B12 and folic acid supplementation (adjusted mean difference: −1.19 μmol/L; 95% CI: −0.09, −2.30). There was no difference in the concentrations of other biomarkers (leptin, adiponectin). Table 3 shows the effects of supplementation with vitamin B12 or folic acid on cardiometabolic risk markers after 6–7 y. There was no difference in the cardiometabolic risk markers between any of the study groups.

**TABLE 2:** Crude and adjusted effects of vitamin B12, folic acid, vitamin B12 + folic acid, and placebo supplementation on cardiometabolic risk markers after 6–7 y^1^

Variables^1^	Leptin (*n* = 274)		HMW adiponectin (*n* = 274)		Total adiponectin (*n* = 274)		Homocysteine (*n* = 776)	
	Unadjusted B coefficient [95% CI]	Adjusted B coefficient^2^ [95% CI]	Unadjusted B coefficient [95% CI]	Adjusted B coefficient^2^ [95% CI]	Unadjusted B coefficient [95% CI]	Adjusted B coefficient^2^ [95% CI]	Unadjusted B coefficient [95% CI]	Adjusted B coefficient^2^ [95% CI]
Placebo (*n* = 67, 202)	Reference							
B12 only group (*n* = 67, 201)	0.30 [−0.87, 1.47]	0.29 [−0.37, 1.16]	−0.48 [−1.00, 0.03]	−0.49 [−1.00, 0.02]	−0.29 [−0.99, 0.41]	−0.29 [−0.99, 0.41]	−0.84 [−1.93, 0.24]	−0.82 [−1.91, 0.26]
Folic acid only (*n* = 70, 204)	−0.07 [−1.23, 1.08]	−0.08 [−1.23, 1.07]	−0.54^3^ [−1.05, −0.03]	−0.54^3^ [−1.05, −0.02]	−0.29 [−0.98, 0.41]	−0.29 [−0.98, 0.41]	−0.74 [−1.81, 0.34]	−0.66 [−1.74, 0.41]
B12 + Folic acid (*n* = 70, 184)	0.15 [−0.62, 0.91]	−0.50 [−1.65, 0.65]	−0.31 [−0.82, 0.20]	−0.31 [−0.82, 0.20]	−0.25 [−0.94, 0.45]	−0.25 [−0.94, 0.45]	−1.18^3^ [−2.29, −0.07]	−1.19^3^ [−2.30, −0.09]

The groups were included in the GLM model with cardiac biomarkers (vitamin B12 only, folic acid only, vitamin B12 + folic acid group, and placebo) after 6–7 y as the dependent variable. GLM, generalized linear model; HMW, high molecular weight. B coefficient, beta coefficient.

1 Leptin and adiponectin were done only in a subsample; homocysteine concentration was measured for all the children in the follow-up.

2 Analysis using GLM with Gaussian family and identity link; adjusted for baseline BMI.

3 Statistically significant at 2-sided *P* < 0.05.

**TABLE 3:** Effect of vitamin B12 compared with no supplementation and folic acid compared with no supplementation on cardiometabolic risk markers after 6–7 y

	Leptin (*n* = 274)		HMW adiponectin (*n* = 274)		Total adiponectin (*n* = 274)		Homocysteine (*n* = 776)	
	Unadjusted B coefficient [95% CI]	Adjusted B coefficent^1^ [95% CI]	Unadjusted B coefficient [95% CI]	Adjusted B coefficent^1^ [95% CI]	Unadjusted B coefficient [95% CI]	Adjusted B coefficent^1^ [95% CI]	Unadjusted B coefficient [95% CI]	Adjusted B coefficient^1^ [95% CI]
No vitamin B12 supplementation					Reference			
B12 supplementation group	−0.07 [−0.88, 0.75]	−0.07 [−0.89, 0.74]	−0.12 [−0.48, 0.24]	−0.12 [−0.48, 0.24]	−0.12 [−0.61, 0.36]	−0.12 [−0.61, 0.37]	−0.63 [−1.41, 0.14]	−0.67 [−1.44, 0.11]
No folic acid supplementation					Reference			
Folic acid supplementation group	−0.43 [−1.25, 0.38]	−0.44 [−1.25, 0.38]	−0.18 [−0.54, 0.17]	−0.18 [−0.54, 0.18]	−0.12[−0.61, 0.36]	−0.12 [−0.61, 0.37]	−0.53 [−1.30, 0.24]	−0.51 [−1.28, 0.26]

To measure the association of supplementation on the cardiac biomarkers after 6–7 y, we reanalyzed the results from our vitamin intervention study, concentrating on the 2 groups of subjects who received folic acid with or without vitamin B12, and vice versa. B coefficient, beta coefficient; HMW, high molecular weight.

1 Analysis using GLM with Gaussian family and identity link; adjusted for baseline BMI.

The mean (SD) and the median (IQR) of the concentrations of cardiometabolic risk markers by intervention and placebo groups during the follow-up are shown in Supplemental Tables 2 and 3.

**FIGURE 2:**
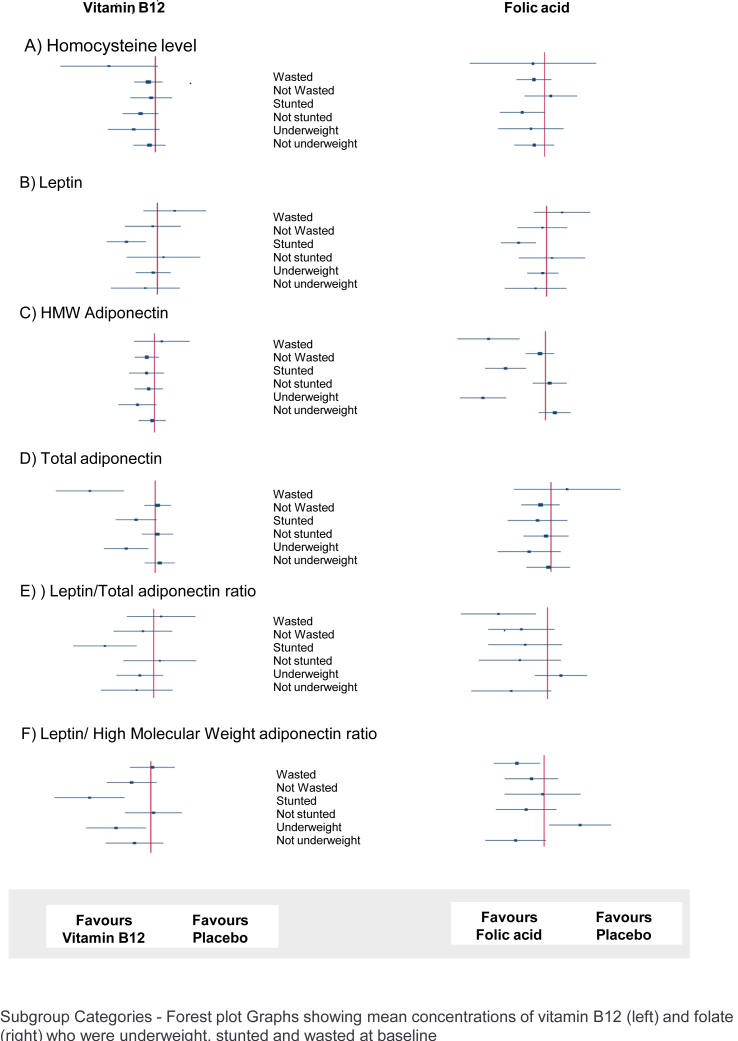
Effect of vitamin B12 or folic acid supplementation on biomarkers based on subgroups wasting, stunting, and underweight on (A) homocysteine concentration, (B) leptin, (C) HMW adiponectin, (D) total adiponectin, (E) leptin/total adiponectin ratio, and (F) leptin/high molecular weight adiponectin ratio. HMW, high molecular weight.

The subgroup analyses (Figure 2) revealed that vitamin B12 supplementation reduced leptin concentration and the LAR as well as LHMWR in stunted children. A similar positive effect was observed in the LHMWR ratio among underweight children. Folic acid supplementation was found to decrease LAR and LHMWR among wasted children. The plasma vitamin B12 and folate concentrations at the follow-up among different supplementation groups are provided in Supplemental Table 4.

## Discussion

The current study is a follow-up of children who participated in a vitamin B12 and/or folic acid supplementation trial during early childhood (6–30 mo of age) to observe the long-term effect of supplementation on cardiometabolic risk markers in later childhood (aged 6–9 y). Supplementation with both vitamin B12 and folic acid for 6 mo in early childhood was associated with a significantly lower tHcy concentration after 6–7 y, which may indicate a synergistic long-term effect. Moreover, analyses conducted on prespecified subgroups of children who were underweight, wasted, or stunted at baseline showed a beneficial effect of supplementing either vitamin B12 or folic acid on leptin and adiponectin concentrations.

Previous cross-sectional observational studies have showed negative associations between vitamin B12 or folate concentrations and cardiometabolic risk markers, especially tHcy concentration in children and adolescents [[28], [29], [30], [31], [32], [33], [34], [35]]. A large community-based cluster-randomized, placebo-controlled, double-blind trial among healthy adults in India found that daily supplementation of 2 or 10 μg of vitamin B12 reduced plasma tHcy concentration, with no additional benefit observed for folic acid supplementation [36]. Studies conducted to determine the association between tHcy and premature cardiovascular disease risk outcomes in children found a modest elevation in tHcy concentrations in those with a family history of CVD risk and elevated systolic blood pressure [16,37,38]. It is challenging to ascertain the clinical significance of these results in children due to the lack of an established threshold for tHcy in the literature. In adults, studies have found that lowering tHcy concentrations by 3 μmol/L reduces risk of ischemic heart disease by 16% [39].

It is interesting to note that early supplementation of vitamin B12 or folic acid in malnourished children had beneficial effects, such as a decrease in leptin concentration and leptin–adiponectin ratio. This decrease is interesting as it is associated with increased insulin sensitivity [[40], [41], [42], [43]]. This novel finding also raises the possibility that malnourished children are more likely to have adverse metabolic outcomes, which are risk factors for adult-life cardiometabolic health, including type II diabetes, according to the Developmental Origins of Health and Disease theory [[3], [4], [5]]. This could have implications for the policy related to micronutrient supplementation in LMICs, as it may lower risk of late-onset CVDs. Further research with larger sample sizes and longer durations of micronutrient supplementation in LMICs should be conducted to confirm these findings.

Risk of vitamin B12 deficiency among young children in India is relatively high due to vegetarianism and prolonged breastfeeding (over 2 y) [44]. It is important to note that AI for this age group is calculated without accounting for vitamin stores at birth and the vitamin B12 concentrations in mother’s milk, both of which are low in vegetarians [45,46]. These factors in combination affect one-carbon metabolism in Indians, who have considerably higher circulating tHcy concentrations than Europeans [47,48]. Previous research has indicated that supplementation with vitamin B12 during preconception and pregnancy had a positive impact on neurodevelopment outcomes at 2 y and vitamin B12 status in early infancy [49,50]. Our findings affirm the life course benefits associated with B-vitamin supplementation in early life, as proposed in previous research.

Given the high risk of developing metabolic diseases in malnourished populations around the world, AI of these vitamins should be ensured to prevent deficiency. Our results show that supplementation of these vitamins in excess of 1 RDA to children at risk of vitamin B12 or folate deficiency could improve their one-carbon metabolism. Stunted and undernourished children might benefit even more from interventions. There is no well-defined safe upper limit for vitamin B12, and for folic acid, it is estimated at 300 μg/d in children between 1 and 3 y of age. Hence, supplementing with >1 AI or RDA is considered safe [51,52]. High doses of folic acid supplementation have been found to impact cellular immunity in animal studies, whereas moderate doses have been known to cause persistent diarrhea in young children [24,53,54]. Although vitamin B12 and folic acid are water-soluble vitamins, it is not unlikely that they could cause adverse effects at higher doses as the dose–response curve for most nutrients may follow an inverted U shape [55].

The major strength of the study is the long-term follow-up of a randomized controlled trial with a retention rate of nearly 80% of the children from the original trial after 6–7 y, with no substantial differences between those included and those who were not [24]. We acknowledge that the likelihood of type I errors could rise with multiple comparisons and a further larger sample size would have improved the validity of our results as few of the biomarkers such as leptin and adiponectin could only be estimated in a subsample. We also recognize that confounding or other biases could have influenced our results. Despite these limitations, we are encouraged by the low rate of missing data in this study.

In conclusion, our long-term follow-up study found that supplementation with vitamin B12 and folic acid in early childhood decreased plasma tHcy concentrations after 6 y. Elevated tHcy is a risk factor for many illnesses, and our findings support supplementing these vitamins in early life. However, whether the reduction of these markers will reduce the CVD risk in adult life needs to be studied using RCTs or cohort studies that follow-up participants into the adult life. In designing such studies, we may need to consider more targeted approaches rather than a “one-size-fits-all” approach.

## Data Availability

Deidentified individual participant data (including data dictionaries) will be made available in addition to the study protocols, statistical analysis plan, and informed consent form. The data will be made available after publication to researchers who provide a methodologically sound proposal for use in achieving the goals of the approved proposal. Proposals should be submitted to ST (e-mail: sunita.taneja@sas.org.in).

## Appendix A Supplementary data

Multimedia component1 Multimedia component1
