# Supplementary material for: Daily Folic Acid and/or Vitamin B12 Supplementation Between 6 and 30 Months of Age and Cardiometabolic Risk Markers After 6–7 Years: A Follow-Up of a Randomized Controlled Trial
Source: J Nutr. 2023 Mar 6;153(5):1493–501. doi: 10.1016/j.tjnut.2023.03.003 (PMC10196576; doi:10.1016/j.tjnut.2023.03.003)
Supplement: Multimedia component1 [file mmc1.docx]

**Supplementary Table 1 Daily vitamin B12 and or folic acid supplementation for 6 months between 6 to 30 months of age and cardiac biomarkers (adipokine) concentrations** **after 6 to 7 years**

| **Cardiac biomarkers** | **Placebo**  **(n=202)** | **Vitamin B12**  **(n=201)** | **Folic acid**  **(n=204)** | **Vitamin B12 and folic acid**  **(n=184)** |
| --- | --- | --- | --- | --- |
| **Total Homocysteine level at follow-up, μmol/L** |  |  |  |  |
| **Mean (SD)**  **Median (IQR)** | 14.3 (6.5)  13.1 (9.9-16.7) | 13.5 (5.4)  12.4 (9.6-15.6) | 13.6 (5.4)  12.4 (9.7-15.9) | 13.1 (4.4)  12.7 (9.9-15.5) |
| **Leptin, ng/L** | **N=67** | **N=67** | **N=70** | **N=70** |
| **Mean (SD)**  **Median (IQR)** | 2.4(3.8)  0.9(0.4 - 2) | 2.7(3.9)  1.2(0.7 – 2.6) | 2.3 (3.2)  1(0.5 – 2.7) | 1.9 (2.7)  0.8(0.5 – 2.1) |
| **HMW Adiponectin, mg/L** |  |  |  |  |
| **Mean (SD)**  **Median (IQR)** | 3.4 (1.8)  3.2(2.2 – 4.3) | 2.9 (1.3)  2.8(2 – 3.9) | 2.9 (1.5)  2.8(1.8 – 3.9) | 3.1 (1.4)  2.9 (2.1 – 3.7) |
| **Total Adiponectin, mg/L** |  |  |  |  |
| **Mean (SD)**  **Median (IQR)** | 7.4 (2.4)  7(5.7 – 8.6) | 7.1 (1.7)  7.1(5.9 – 8.2) | 7.1 (2.1)  6.9 (5.6 – 8.4) | 7.2 (2)  6.8 (5.9 – 8.5) |
| **Leptin/HMW Adiponectin** |  |  |  |  |
| **Mean (SD)**  **Median (IQR)** | 1 (1.8)  0.3 (0.2-1.1) | 1.2 (2)  0.4(0.2-1.2) | 1.2 (2.1)  0.4(0.2-1.2) | 0.6 (0.9)  0.3 (0.2-0.8) |
| **Leptin/Total Adiponectin (LAR)** |  |  |  |  |
| **Mean (SD)**  **Median (IQR)** | 0.4 (0.6)  0.1 (0.1-0.3) | 0.4 (0.6)  0.1(0.1-0.4) | 0.4 (0.5)  0.1(0.1-0.5) | 0.3 (0.3)  0.1(0.1 – 0.3) |

**Supplementary table 2 Daily vitamin B12 supplementation for 6 months between 6 to 30 months of age and cardiac biomarkers (adipokine) concentrations compared to no B12 supplementation** **after 6 to 7 years**

| **Cardiac biomarkers** | **Vitamin B12 group**  **(n=385)** | **Non B12 group**  **(n=406)** |
| --- | --- | --- |
| **Total thcy at follow-up, μmol/L** |  |  |
| **Mean (SD)** | 13.3 (4.9) | 13.9 (5.9) |
| **Median (IQR)** | 12.5 (9.8-15.6) | 12.7 (9.8-16.2) |
| **Leptin, ng/L** |  |  |
| **Mean (SD)** | 2.3 (3.4) | 2.3 (3.5) |
| **Median (IQR)** | 0.9 (0.6-2.4) | 0.9 (0.5-2.1) |
| **HMW Adiponectin, mg/L** |  |  |
| **Mean (SD)** | 3 (1.4) | 3.2 (1.7) |
| **Median (IQR)** | 2.8 (2.1-3.8) | 2.9 (1.9-4) |
| **Total Adiponectin (mg/L)** |  |  |
| **Mean (SD)** | 7.1 (1.9) | 7.3 (2.3) |
| **Median (IQR)** | 6.9 (5.9-8.3) | 7 (5.7-8.5) |
| **Leptin/HMW Adiponectin** |  |  |
| **Mean (SD)** | 0.9 (1.5) | 1.1 (1.9) |
| **Median (IQR)** | 0.3 (0.2-0.9) | 0.3 (0.2-1.1) |
| **Leptin/Total Adiponectin (LAR)** |  |  |
| **Mean (SD)** | 0.3 (0.5) | 0.4 (0.5) |
| **Median (IQR)** | 0.1 (0.1-0.3) | 0.1 (0.2-0.3) |

HMW Adiponectin- High Molecular weight Adiponectin

**Supplementary Table 3 Daily folic acid supplementation for 6 months between 6 to 30 months of age on the cardiac biomarkers (adipokine) concentrations compared to no folic acid supplementation at 6 – 9 years of life**

| **Cardiac biomarkers** | **Folic acid group**  **(n=388)** | **Non-folic acid group**  **(n=403)** |
| --- | --- | --- |
| **Thcy at follow-up, μmol/L** |  |  |
| **Mean (SD)** | 13.4 (4.9) | 13.8 (6) |
| **Median (IQR)** | 12.5 (9.9-15.7) | 12.7 (9.7-16.2) |
| **Leptin, ng/L** |  |  |
| **Mean (SD)** | 2.1 (2.9) | 2.5 (3.9) |
| **Median (IQR)** | 0.9 (0.5-2.6) | 1 (0.6-2.3) |
| **Adiponectin HMW, mg/L** |  |  |
| **Mean (SD)** | 3 (1.5) | 3.2 (1.6) |
| **Median (IQR)** | 2.8 (2.1-3.8) | 3 (2-4) |
| **Adiponectin Total, mg/L** |  |  |
| **Mean (SD)** | 7.1 (2.1) | 7.3 (2.1) |
| **Median (IQR)** | 6.8 (5.8-8.4) | 7.05 (5.8-8.4) |
| **Leptin/HMW Adiponectin** |  |  |
| **Mean (SD)** | 0.9 (1.7) | 1.1 (1.9) |
| **Median (IQR)** | 0.4 (0.2-0.9) | 0.3 (0.2-1.1) |
| **Leptin/Total Adiponectin (LAR)** |  |  |
| **Mean (SD)** | 0.3 (0.5) | 0.4 (0.6) |
| **Median (IQR)** | 0.1 (0.1-0.3) | 0.2 (0.1-0.3) |

HMW Adiponectin- High Molecular weight Adiponectin**Supplementary Table 4. Mean (SD) plasma concentrations of vitamin B12 or folic acid supplementation group in subgroups at follow-up (6 to 9 years of age)**

|  | **Plasma B12 (pmol/L), mean (SD) in B12 supplementation group** | **Plasma B12 (pmol/L), mean (SD) in no B12 supplementation group** | **Plasma folate (nmol/L), mean (SD) in folic acid supplementation group** | **Plasma folate (nmol/L), mean (SD) in no folic acid supplementation group** |
| --- | --- | --- | --- | --- |
| **Stunted** | N= 44 | N=50 | N= 49 | N=45 |
|  | 326.9 (152.2) | 308.3 (153.1) | 18.7 (9.4) | 20.6 (11.4) |
| **Underweight** | N= 33 | N=45 | N= 44 | N=34 |
|  | 310.2 (152.7) | 294.2 (143.6) | 18.7 (9.8) | 16.8 (5.7) |
| **Wasted** | N= 17 | N=14 | N= 14 | N=17 |
|  | 259.4 (80.1) | 248.9 (103.6) | 18.8 (13.3) | 17.7 (6.3) |
